# Denning coyotes (*Canis latrans*) select fine-scale habitat characteristics that promote den stability in a protected area

**DOI:** 10.1093/jmammal/gyag072

**Published:** 2026-08-19

**Authors:** Sage Raymond, Juno Montgomery, Colleen Cassady St. Clair

**Affiliations:** Department of Biological Sciences, University of Alberta, 116 St & 85 Ave, Edmonton, Alberta, T6G 2R3, Canada; Department of Biological Sciences, University of Alberta, 116 St & 85 Ave, Edmonton, Alberta, T6G 2R3, Canada; Department of Biological Sciences, University of Alberta, 116 St & 85 Ave, Edmonton, Alberta, T6G 2R3, Canada

**Keywords:** *Canis latrans*, Coyote, den selection, ecology, habitat selection, protected area, área protegida, *Canis latrans*, coyote, ecología, selección de hábitat, selección de madriguera

## Abstract

Den selection is critical to reproductive success and offspring survival in many mammal species, but little is known about den site selection by wild coyotes (*Canis latrans*) outside of urban contexts. We examined the third- and fourth-order habitat characteristics of 42 Coyote dens in Elk Island National Park, a protected natural area in central Alberta, Canada. Using resource selection functions, we evaluated 3 hypotheses: that coyotes select dens based on (i) physical den suitability; (ii) security from predators, people, or dogs; and (iii) access to food and water resources. Coyotes exhibited strong fourth-order selection for dens with cohesive, inorganic soils and steep slopes that promote structural integrity and drainage. Structural supports such as roots and abandoned but persisting Beaver (*Castor canadensis*) infrastructure were common. We found limited evidence of selection for features that promoted security, contrasting other studies that found security to be an important predictor of den sites. We found no evidence for selection based on local resource availability. Our findings indicate that fine-scale habitat features related to den stability are the primary drivers of den site selection in wild coyotes in this ecosystem and suggest that den selection is flexible and context-dependent in response to local environmental pressures.

## Resumen

La selección de madrigueras es crucial para el éxito reproductivo y la supervivencia de las crías en muchas especies de mamíferos, pero se sabe poco sobre la selección de madrigueras por parte de los coyotes salvajes (*Canis latrans*) fuera de los entornos urbanos. Examinamos las características de hábitat de tercer y cuarto orden de 42 madrigueras de coyotes en el Parque Nacional Elk Island, un área natural protegida en el centro de Alberta, Canadá. Mediante funciones de selección de recursos, evaluamos tres hipótesis: que los coyotes seleccionan madrigueras en función de (1) la idoneidad física de la madriguera, (2) la seguridad frente a depredadores, personas o perros, y (3) el acceso a recursos alimenticios y hídricos. Los coyotes mostraron una fuerte selección de cuarto orden por madrigueras con suelos cohesivos e inorgánicos y pendientes pronunciadas que favorecen la integridad estructural y el drenaje. Los soportes estructurales, como raíces e infraestructura de castores (Castor canadensis) abandonada pero persistente, eran comunes. Encontramos cierta evidencia de selección por características que promovían la seguridad, en contraste con otros estudios que encontraron que la seguridad es un predictor importante de la ubicación de las madrigueras. No encontramos evidencia de selección basada en la disponibilidad de recursos locales. Nuestros hallazgos indican que las características del hábitat a pequeña escala relacionadas con la estabilidad de las madrigueras son los principales factores que influyen en la selección del lugar de construcción de las madrigueras en los coyotes salvajes de este ecosistema, y sugieren que dicha selección es flexible y depende del contexto en respuesta a las presiones ambientales locales.

Successful denning is critical to the life history of various mammals, with impacts on offspring survival and population dynamics (Ross et al. 2010; van der Meer et al. 2013). Dens protect vulnerable and altricial offspring from the elements and predators while providing a secure place that supports the energy conservation needs of lactating females. For example, polar bears (*Ursus maritimus*) use dens to have a protected area to give birth during hibernation, which helps them conserve fat (Robbins et al. 2012). Den selection by carnivores is influenced by various factors. Denning animals often select sites with environmental features that support den integrity and thermoregulation (Ross et al. 2010). Additionally, animals may select sites with a low density of competitors and predators (Ross et al. 2010; van der Meer et al. 2013) and at hidden or secluded sites (Trapp et al. 2008; Elbroch et al. 2015), presumably to increase security for offspring (Robbins et al. 2012). Subordinate carnivores select dens that provide hunting opportunities while avoiding detection from dominant carnivores (Punjabi et al. 2013; Lendrum et al. 2014). Some carnivore species perceive humans as predators (Frid and Dill 2002) and therefore avoid denning near human infrastructure (Van Dyke et al. 1986; Sahlén et al. 2011). Finally, resource availability influences den selection; many carnivores select dens in areas of high prey availability (Nellis and Keith 1976; Grigione et al. 2002) or with easy access to water (Trapp et al. 2008). Because dens play a crucial role in offspring survival and parental fitness, understanding den characteristics can promote the protection of sites that foster the persistence of local populations (Fernández and Palomares 2000; Pigeon et al. 2016).

Many species select resources at different scales (Johnson 1980). Dens are selected within the home range (third-order) and for microhabitat features (fourth-order). For example, grizzly bears (*U. arctos*) exhibit strong third-order selection at dens for features such as high-quality spring food and concealment cover (Pigeon et al. 2016). Many carnivores select den characteristics at the fourth-order. For example, Pallas’s cats (*Otocolobus manul*) select den sites that promote thermoregulation with shade in the summer and insulation in the winter (Ross et al. 2010), and cougars (*Puma concolor*) select sites at highly concealed and protected microsites to promote offspring security (Elbroch et al. 2015). Similarly, African wild dogs (*Lycaon pictus*) select dens with little visibility that provide optimal protection for their offspring (van der Meer et al. 2013). Some species exhibit fourth-order selection for den sites that have been previously excavated by other species or individuals, especially beavers (Scrafford et al. 2020), which suggests greater den stability and presumably lessens energetic expenditure in excavating a new den. In North America, third and fourth-order habitat selection at den sites has been described for canids, including wolves (*Canis lupus*; Trapp et al. 2008), red foxes (*Vulpes vulpes*; Paynter et al. 2024), and urban-dwelling coyotes (*Canis latrans*; Raymond and St. Clair 2023a). Differences in canid den selection at varying levels of urbanization are lacking.

Coyotes are ubiquitous mesocarnivores that occupy various environments across North America, including urban areas (Gompper 2002). Over the last 200 yr, a lack of predators, increased food availability, and behavioral flexibility have enabled Coyote range expansion (Gehrt and Riley 2010; Breck et al. 2019). Despite their ubiquity, knowledge of Coyote den selection is limited. Most existing studies employ small sample sizes and have been geographically restricted to the United States (Althoff 1980; Harrison and Gilbert 1985; Way et al. 2001). Coyotes may establish dens that promote thermoregulation by having southeastern exposure (Harrison and Gilbert 1985), although one study found that protection from wind was more important than insolation (Althoff 1980), and another found no selection for aspect (Way et al. 2001). Often, Coyote dens are excavated from other animal burrows such as badgers (*Taxidea taxus*; Althoff 1980) and are dug into soft soils like loam that reduce energetic expenditure of digging (Althoff 1980; Harrison and Gilbert 1985). Coyotes may also establish dens that use tree roots as important structural components that promote den integrity (Way et al. 2001). Studies of Coyote den selection from cities suggest that animals incorporated anthropogenic features into dens, such as denning under decks, buildings, and refuse (Way 2009; Raymond and St. Clair 2023a). A study of urban Coyote den selection that used a large sample size (*n *= 120 dens) in the city of Edmonton concluded that coyotes selected more strongly at the fourth-order of habitat selection, especially for features related to security from potential disturbance by people and dogs (Raymond and St. Clair 2023a), but it is unclear if coyotes select similar traits at similar scales in natural areas.

Our objective was to determine third- and fourth-order habitat characteristics selected by coyotes at dens in a natural area in the Elk Island National Park. This site is near Edmonton, Canada, where a previous study of urban den sites occurred. This proximity allowed a comparison of Coyote den site selection in natural and urban settings (Raymond and St. Clair 2023a). To do so, we assessed support for 3 broad, non-mutually exclusive hypotheses (Table 1). First, we hypothesized that coyotes select dens based on physical features that promote den stability and thermoregulation, predicting selection for warm, steep, sunny, dry locations with stable soils. Second, we hypothesized that coyotes would select features that promote security from people and predators. Finally, we hypothesized that coyotes may select sites with high local resource availability, predicting that they would select dens near water and productive hunting or foraging areas. We aimed to provide insight into the habitat characteristics that coyotes select at den sites and the scales at which they do so in the protected natural area of Elk Island National Park.

**Table 1:** Hypotheses and predictions related to Coyote (*Canis latrans*) den selection in Elk Island National Park, Canada.

Hypothesis	Explanation	Predictions
**(1) Den suitability and stability**	Coyotes select existing dens and dens that are unlikely to collapse. To promote pup survival, coyotes choose dens that will keep pups warm and dry.	Dens will: (a) Have stable, cohesive, inorganic soils (b) Include structural support (e.g., rock, tree root) (c) Be previously excavated by another organism (d) Have a southern aspect (e) Have a steep slope to promote drainage
**(2) Security**	Coyotes select dens that protect vulnerable pups from predation and disturbance. To promote security, coyotes choose dens away from human infrastructure and at sites with vegetation and topography that limit detection	Dens will: (a) Be in secluded areas where vegetative cover types meet and far from other dens (b) Be far from the park boundary, roads, and other human infrastructure and at sites with low road density (c) Have high canopy and hiding cover (d) Occur on steep slopes
**(3) Resources**	Coyotes select dens near abundant water and foraging opportunities, which allows parents to expend less energy while foraging and remain near the den, where they could defend pups.	Dens will: (a) Be closer to water (b) Have higher cover by water (c) Be located near areas with abundant open meadow habitat

## Methods

### Study area

Data were collected from Elk Island National Park (EINP), located ∼40 km east of Edmonton (53.578786, −112.838318) in the Beaverhills United Nations Educational, Scientific, and Cultural Organization Biosphere Reserve. Covering a total area of ∼196km^2^, EINP is divided by a highway into 2 sections; the main park (134 km^2^) is north of Highway 16, and an additional isolation area for ungulate management (60 km^2^) is to the south. A 2.2 m page wire fence fully encloses these areas to separate large ungulates, especially Bison (*Bison bison*), from the highway and the agricultural lands surrounding the park (Pruss 2002). The park manages large populations of ungulates, including Plains and Wood Bison (*B. bison bison*, *B. bison athabascae*), White-tailed Deer (*Odocoileus virginianus*), and Elk (*Cervus elaphus*; Foca and Boyce 2022). Predators are limited in EINP, with coyotes (*C. latrans*) and black bears (*U. americanus*) being the most common, and rare reports of wolves (*C. lupus*) and lynx (*Lynx canadensis*; Foca and Boyce 2022). The ecozone is classified as aspen parkland and is located at the transition between prairie to the south and boreal forest to the north (Pruss 2002). EINP includes diverse and complex plant communities that provide forage, protection, and thermal cover (Bork et al. 1997). The park has stands of trees, a prolific shrub layer, and open areas dominated by grasses (Bork et al. 1997). The region has long, cold winters, often with deep snow for approximately 4 to 5 mo in most years (mid-November through mid-April) and moderate, short ­summers with seasonal flooding of ephemeral, sedge-dominated meadows and lowlands in the spring (Hood 2007).

### Den detection

We detected dens by following wildlife trails with visible Coyote tracks in September 2023 (Raymond and St. Clair 2023a). Although obligate use of dens is restricted to spring, coyotes visit and use both natal (i.e., dens in which pups had been born) and non-natal dens throughout the calendar year (Raymond 2023; authors’ personal observations). This pattern allowed us to be confident in den detection, while performing surveys at a time of year that would not disturb animals during the most sensitive periods, and which aligned with the restrictions of our animal care and park use permits. Because EINP has high densities of coyotes but little is known about their denning habits, we targeted areas suggested by park staff and areas with vegetation that suggested dry soils near the center of known Coyote home ranges (Pruss 2002). We followed wildlife trails with evidence of use by coyotes, which we assessed based on the presence of Coyote tracks (Elbroch and McFarland 2019). Upon detecting a wildlife trail, we began recording tracks using GPS. If we inadvertently lost the wildlife trail, or if the trail stopped, we paused GPS recording. While walking, we visually searched for dens. We classified cavities as dens if they met the following 3 criteria: (i) within the published dimensions of Coyote dens (Way et al. 2001; Elbroch and McFarland 2019; Raymond and St. Clair 2023a); (ii) greater than 1.5 m deep; and (iii) associated with either a throw mound of disturbed soil or Coyote hairs around the entrance (Way et al. 2001). While all dens were large enough to accommodate adult coyotes, we could not confirm whether dens were natal dens.

### Third-order habitat characteristics

For each of the 42 dens that we identified, we generated 5 available sites by randomly generating 210 points constrained along the paths walked while searching for dens (Manly et al. 1993; Boyce and McDonald 1999). Although many studies generate available points randomly within known home ranges based on collar data (e.g., Elbroch et al. 2015), no coyotes were collared in the region at the time of our study. We selected random sites along paths because these sites represented areas that coyotes had travelled, ensuring they could be considered available for use (Raymond and St. Clair 2023a; Boyers and Parrini 2024). We then extracted landscape variables at used and available points (Manly et al. 1993; Boyce and McDonald 1999; Raymond and St. Clair 2023a). We used GIS to measure Euclidean distance (m) to the nearest known den. We built a digital elevation model using elevation data extracted from Google Earth to determine elevation (m), aspect (°), and slope (%) at all sites (25 m resolution). We converted aspect to a continuous East Index by taking the sine of the aspect in radians, resulting in a variable that ranged from −1 (west) to 1 (east). We created a South Index by taking the cosine of the aspect in radians and multiplying by negative 1, resulting in a variable that ranged from −1 (north) to 1 (south; Raymond and St. Clair 2023a). All GIS analyses were performed using the “terra” package (Hijmans et al. 2023) in R (R Core Team 2025).

We used the province of Alberta’s open-source mapping to determine the distance to the nearest road and park boundary (m), and to calculate road density at plot center in m/km^2^ at a 25 m resolution (Government of Alberta 2022). We calculated the distance to water, trees, and open areas (including shrubs; m) using the Alberta Vegetation Inventory (AVI; Government of Alberta 2022). We also used the AVI database to calculate the vegetation edge density by drawing a line around the border of all forested stands (6% to 100% crown closure) and calculating the edge density (m/km^2^) at a 25 m resolution. We followed the same steps for open areas, which consisted of non-forested (<6% tree cover) terrestrial areas. These metrics are potentially influential for Coyote den selection because animals adapted to open environments rely on forested habitat to evade predators and open habitat for early detection of threats. To explore the influence of land cover within different distances of dens, we generated a series of buffers around points with a radius of 12.5, 25, 50, 100, 250, and 500 m; within each buffer, we calculated the percent cover by trees, water, and open habitat. We tested the performance of all distance metrics as decay terms using the formula decay distance = 1-(e^-αd^), where α is the decay term and d is the Euclidean distance to the feature (m; Nielsen et al. 2009; Malcolm et al. 2020). We tested a broad range of α values (0.002, 0.004, 0.006, 0.012, 0.03, 0.06, and 0.2) and proceeded with the term that resulted in the lowest Akaike information criterion adjusted for small sample size (AIC_c_) in a univariate model.

### Fourth-order habitat characteristics

We measured fourth-order habitat characteristics in August and September 2024, 1 yr after detecting dens, which allowed us to assess characteristics in summer conditions without risk of disturbing animals during biologically sensitive periods (Raymond and St. Clair 2023a). We replicated the methods used by Raymond and St. Clair (2023a) at Coyote dens in urban sites. At each den, we counted the number of entrances and measured the height and width of all openings. We established a plot center in the middle of the largest den entrance. We established a single paired plot per use site (Klaassen and Broekhuis 2018; Schweiger et al. 2021). To do so, we used a random number generator to generate an azimuth and walked 50 m (Raymond and St. Clair 2023a). If the resulting point occurred in water, we generated additional random bearings until the plot center occurred on land. We recorded the structural support of each den. After noticing evidence of past Beaver (*C. canadensis*) activity such as excavations, and sticks at several dens, we began recording this information systematically within 5 m of the plot center.

We measured fourth-order habitat characteristics at each den and paired site. From the plot center, we used clinometers to measure slope (%) both 2 m above and 2 m below the plot center and later calculated the average slope. We measured the aspect (°) of the slope upon which the den or paired site was located using a compass, except at sites on flat ground, which had no aspect (Province of British Columbia 2010). To preserve sample size, when use and available sites had no aspect, we later substituted the mean aspect of all used and available sites, respectively. We developed an East and South Index as described for the third-order analysis. We measured canopy cover (%) using a densiometer from a height of 50 cm at 5 points: plot center, 2 m above and below plot center, and 2 m to the right and left of plot center (Pigeon et al. 2016), parallel to the slope. Doing so quantified the extent to which the area was covered by all vegetation >50 cm in height. We measured hiding cover (%) by observing a 90 cm cover pole positioned at the 4 latter positions from the plot center (Trapp et al. 2008). Hiding cover can be provided by any vegetation type, or by debris or topography. When dens had multiple entrances we measured slope, canopy cover, and hiding cover measurements relative to the outermost den opening in each direction. For each site, we calculated the variance of the 4 hiding cover and the 5 canopy cover measurements. We also calculated the difference between the maximum and minimum measures of hiding cover and canopy cover. We collected a single soil sample from the plot center of all used and available sites. We used standard soil classification techniques (Province of British Columbia 2010) to classify soil texture at plot center. We used standard methodology to create a “soil worm” by moistening soil and rolling the substrate in our fingers (Province of British Columbia 2010). We recorded the diameter (mm) of the smallest soil worm that could be created. Smaller diameters indicate stabler, more cohesive soils (Province of British Columbia 2010). Within a 5 m radius of the den, we estimated the percent cover for each of trees (i.e., woody species >2 m in height), shrubs (i.e., woody species <2 m in height), herbaceous plants (i.e., non-woody plant species), bare ground, and water (Province of British Columbia 2010).

### Analysis

For both third and fourth-order habitat variables, we explored habitat selection using resource selection functions (RSFs) that compared characteristics of used and available sites (Boyce and McDonald 1999). We did not test a suite of mutually exclusive a priori hypotheses because multiple mechanisms often influence complex ecological process like den selection, and because developing repeatable and biologically meaningful a priori hypotheses is challenging when addressing questions or systems for which little existing information exists (Tredennick et al. 2021). We instead limited the risk of spurious results by using an iterative variable selection procedure that ensured each covariate was biologically and statistically meaningful (Hosmer and Lemeshow 2000). To reduce the risk of overfitting, we excluded predictors that exhibited limited variation across the dataset (Dormann et al. 2013). Specifically, after scaling and centering data, we calculated the interquartile range for each predictor and eliminated those associated with range <0.1 (Kuhn 2008). We then classified used sites as 1 and available sites as 0 and used the “lme4” package (Bates et al. 2015) to perform logistic regression to generate univariate models that tested the performance of each predictor as both a linear and a quadratic term; we retained those with liberal significance (*P *< 0.25) for subsequent modelling (Hosmer and Lemeshow 2000). We included predictors as quadratic terms because selection or avoidance of intermediate values is common in ecology. Predictors were retained as quadratic terms if the quadratic model lowered the AIC_c_ by ≥ 2 and if the quadratic term was liberally significant (*P *< 0.25). We also used univariate models to select the most predictive decay term for each distance variable by retaining the term with the lowest AIC_c_ (Nielsen et al. 2009; Malcolm et al. 2020). We then used the package “MuMIn” (Barton 2016) to complete all subsets modelling (Eberhardt 2003; Hegyi and Garamszegi 2011; Tredennick et al. 2021), but we did not develop models with correlated predictors (*r *> 0.6; Dormann et al. 2013). We selected models within ΔAIC_c_ ≤ 2, assuming they had equal predictive power (Hosmer and Lemeshow 2000). We then averaged these models (conditional) and reported standardized coefficients (Symonds and Moussalli 2011). We emphasized effect sizes by calculating odds ratios. We assessed model performance using the “performance” package (Lüdecke et al. 2021). Variance inflation factors were calculated for the final models to ensure the absence of multicollinearity. We calculated the area under receiver operator characteristic (AUC ROC) curves to quantify model predictive accuracy (Cumming 2000) and Nagelkerke and McFadden pseudo *R^2^* values to indicate model fit (Veall and Zimmermann 1994). All analyses were performed using R version 4.5.1 (R Core Team 2025).

## Results

In September 2023, we detected 42 Coyote dens while following 313 kilometers of wildlife trails. In August and September 2024, we completed habitat surveys at each den and paired available site. Most dens had a single opening (*n *= 32, 76.2%); 5 had 2 entrances, and 3 had 3 entrances. The openings of 2 dens had collapsed between detection in 2023 and follow-up surveys in 2024, preventing measurement. Both were located at sites with sandy soil and did not have structural support. Dimensions of the remaining den openings, including dens with multiple entrances, were 26.8 ± 3.7 cm high and 29.1 ± 5.2 cm wide. Most dens occurred in silt clay soil (*n *= 14, 33%) and silt clay loam (*n *= 7, 17%), with 6 other soil types found across all sites. No dens were in organic soil, but 28 (67%) of the available sites were. Most den openings (*n *= 48, 96%) incorporated structural support, including tree or shrub roots (*n *= 40, 80% of supported dens), old Beaver infrastructure (lodge, channel, bank burrow; *n *= 23, 46%), or rocks (*n *= 4, 8%). Because it was not originally part of the predictors we selected a priori, we chose not to include presence of Beaver infrastructure in our modelling approach, but we quantified that roughly half of the dens (*n *= 22, 52.4%) were within 1 m of Beaver-chewed tree stumps (Fig. 1), while only 11 (26.2%) of available sites were.

**Fig. 1:**
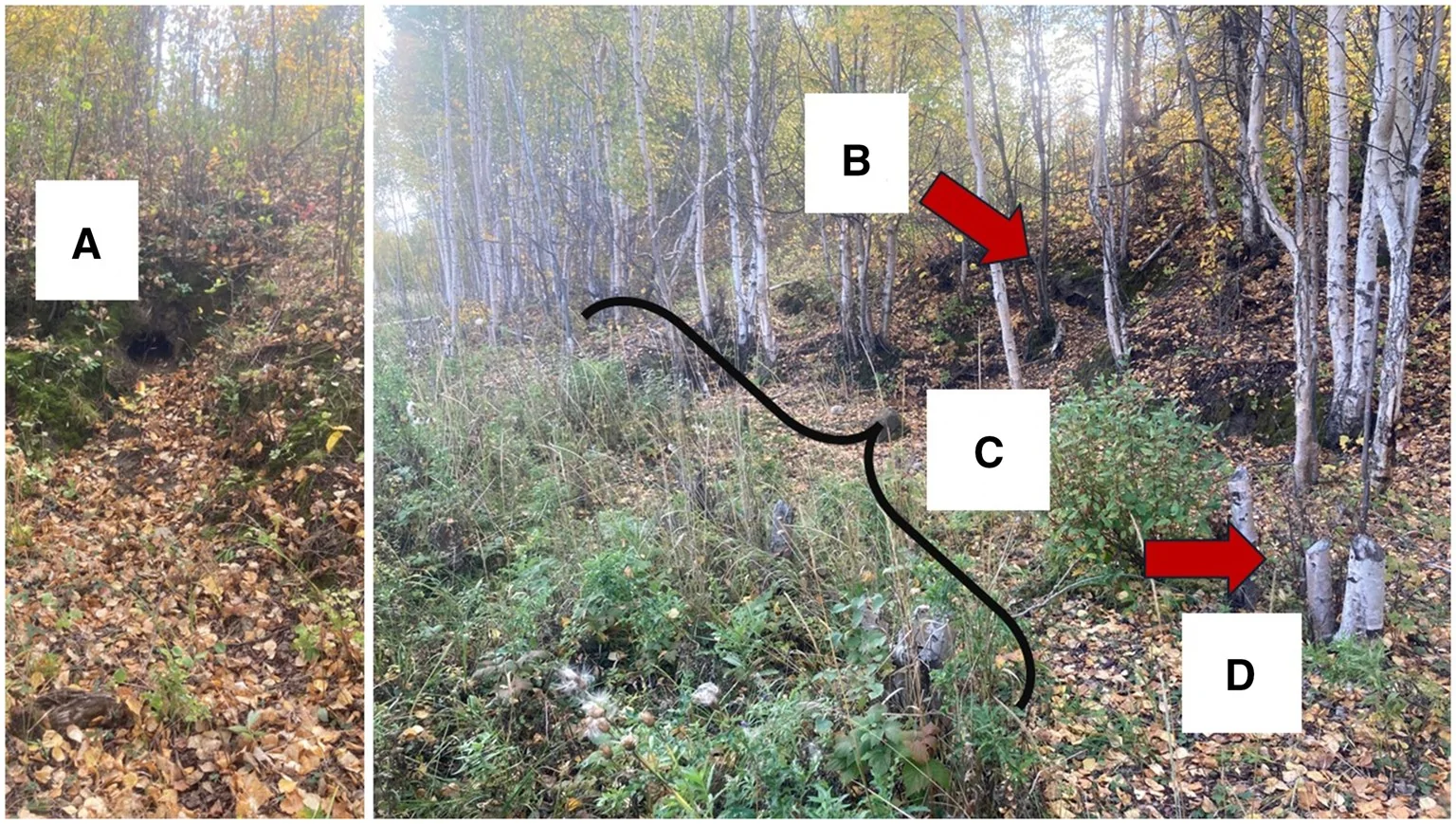
Evidence of prior beaver use at Coyote (*Canis latrans*) dens in Elk Island National Park, Alberta, Canada, 2024. Coyote dens often occurred at the entrance of old Beaver (*Castor canadensis*) excavations (A, B). Changes in the vegetation such as this edge between a forb-dominated, flat expanse, and a treed hillside show that this area was previously a pond (C). Beaver-chewed sticks and trees (D) were often present near den sites, but rarely near available sites.

To explore third-order habitat selection, we developed RSFs using all 42 dens and 210 available sites (Table 2). After testing distance metrics as decay terms, we found that distance to den, water, road, and park boundary performed best with α = 0.04, 0.06, 0.006, and 0.06, respectively (Supplementary Data SD1). The list of variables retained after exclusion of covariates with low variation and univariate modeling included distance to trees (m) and open areas (m), East and South Indexes, slope (%), open edge density, road density, and percent cover by water, open areas, and trees within a 250, 50, and 100 m buffer, respectively. We also retained decay terms for den, park boundary, and water (Supplementary Data SD1). Of these variables, percent tree and open cover, and road density performed best using a quadratic model (Supplementary Data SD2). Correlated terms (*r *> 0.6) included distance to trees (m) and distance to open areas (m), and percent water cover within a 50 m buffer and the decay term for distance to water.

**Table 2:** Summary metrics for the conditional averaged results of the top 12 models explaining third-order characteristics of Coyote (*Canis latrans*) den sites in Elk Island National Park, Alberta, Canada, 2024. Metrics reported include standardized beta coefficients (*β*) and associated *P* values (*P*), odds ratio (OR), and upper and lower confidence intervals (CI) for both beta coefficients and odds ratios.

Variable	***β***	*P*	Low CI	Upp CI	OR	OR Low CI	OR Upp CI
**Decay Dist. to Den**	0.719	<0.001	0.353	1.085	14.561	3.724	56.937
**Open cover (%)**	0.746	0.036	0.049	1.443	1.102	1.017	1.195
**Open cover (quadratic; %)**	−0.436	0.035	−0.841	−0.030	0.998	0.997	1.000
**Tree cover (%)**	0.766	0.102	−0.153	1.685	0.875	0.789	0.969
**Tree cover (quadratic; %)**	1.115	0.005	0.342	1.888	1.001	1.000	1.002
**Water cover (%)**	−0.387	0.094	−0.841	0.067	0.977	0.952	1.004
**Road density (m/km^2^)**	0.373	0.178	−0.169	0.914	1.591	0.810	3.126
**Slope (%)**	0.316	0.189	−0.155	0.787	1.942	0.721	5.225
**Dist. to trees (m)**	1.460	0.259	−1.075	3.996	1.027	0.980	1.076
**East index**	0.258	0.213	−0.148	0.664	1.448	0.808	2.595
**Decay Dist. to boundary**	−0.423	0.217	−1.095	0.249	0.136	0.006	3.237

The all-subsets modeling approach originally resulted in dozens of models within 2 AIC_c_. To restrict inference to models with the most support and to avoid inclusion of models with comparatively weak empirical support that can arise when using broader thresholds in large candidate sets, we retained only models within 0.5 AIC_c_, resulting in 12 models (Fig. 2; Table 3). Model-averaged results suggested that the strongest predictor of denning was greater distance to the nearest den (*β*  =  0.719, *P *< 0.001); odds of a den increased from ∼35% 50 m from a den to ∼80% at a distance of 400 m (Fig. 2B). Coyotes also selected sites with lower percent cover by water within a 50 m buffer (*β* = −0.387, *P *= 0.094). Each 10% increase in water cover reduced odds of a den by 9.3% (Fig. 2C). Coyotes selected against sites with intermediate tree cover within a 100 m buffer (Fig. 2D), and selected for sites with intermediate levels of open cover (Fig. 2E). Several other predictors were included in the final model results, but all had confidence intervals that considerably overlapped zero (Fig. 2A). A maximum variance inflation factor of 1.72 suggested that models had limited multicollinearity. The average Area Under the Curve Receiver Operator Characteristic (AUC ROC) of 0.87 suggested that the models discriminated well between used and available sites. Moderate Nagelkerke (0.445) and McFadden (0.341) pseudo *R^2^* values indicated that the models had a relatively poor fit.

**Fig. 2:**
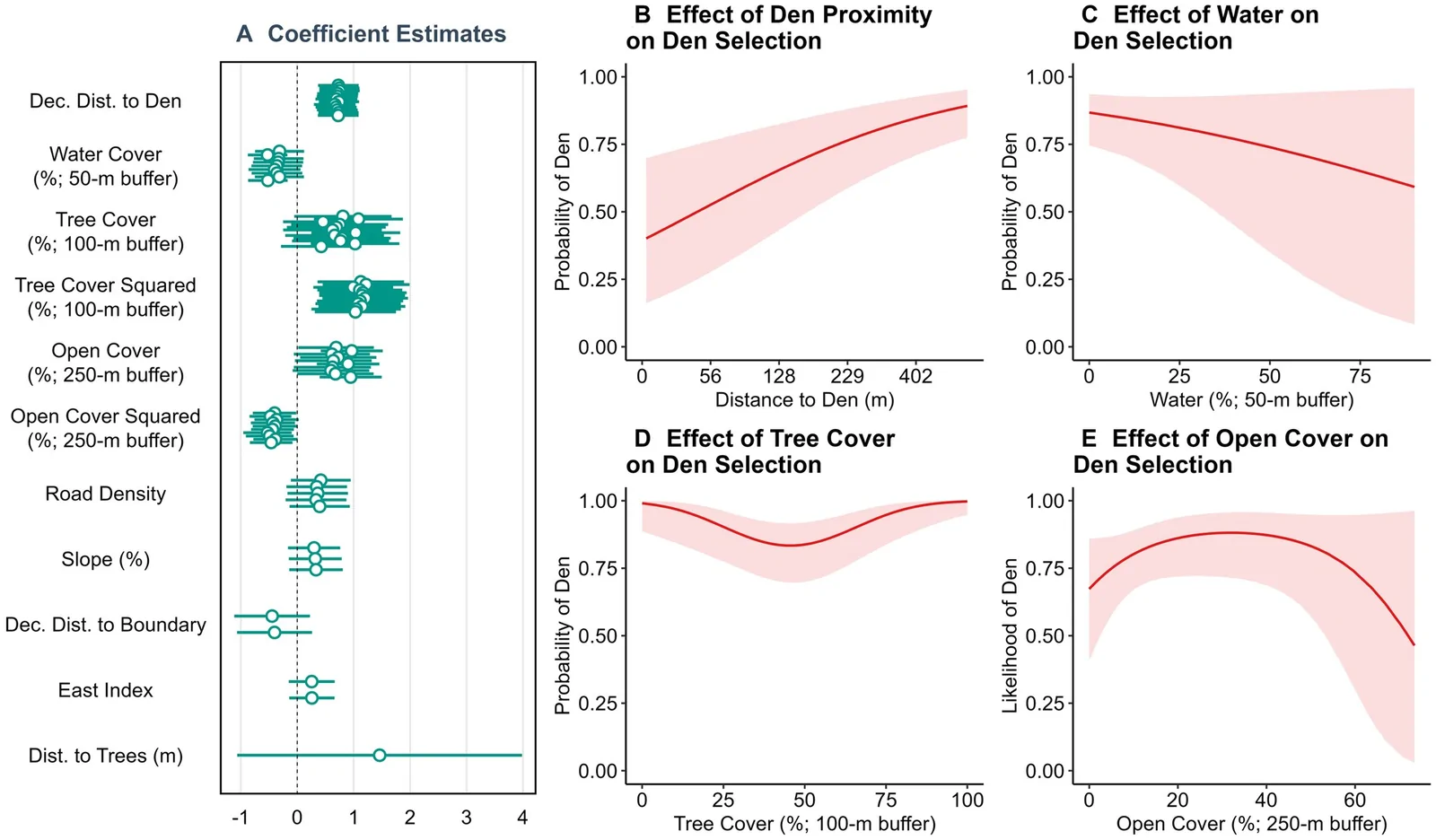
Standardized third-order parameter estimates and 95% confidence intervals for all models within 0.5 Akaike Information Criterion adjusted for small sample sizes including key variables selected by coyotes (*Canis latrans*) for den sites in Elk Island National Park, Alberta, Canada, 2024. (A) Coefficient estimates including 95% confidence intervals, ordered by frequency of occurrence in all models. (B) Effect of distance to nearest den (m) on den likelihood. (C) Effect of percent water cover within a 50 m buffer on den likelihood. (D) Effect percent tree cover within a 100 m buffer on den likelihood. (E) Effect of percent open cover within a 250 m buffer on den likelihood.

**Table 3:** Conditional model-averaged results for the top 4 models explaining fourth-order characteristics selected by coyotes (*Canis latrans*) at den sites and available sites in Elk Island National Park, Alberta, Canada, 2024. Metrics reported include standardized beta coefficients (*β*) and associated *P* values (*P*), odds ratio (OR), and upper and lower confidence intervals (CI) for both beta coefficients and odds ratios.

Variable	***β***	*P*	Low CI	Upp CI	OR	OR Low CI	OR Upp CI
**Avg. slope (%)**	3.373	<0.001	1.514	5.232	1.211	1.090	1.345
**Shrub cover (%)**	−1.342	0.025	−2.513	−0.170	0.965	0.920	0.994
**Herb cover (%)**	1.254	0.027	0.146	2.363	1.040	1.005	1.076
**Min. Soil diameter**	−1.765	0.032	−3.381	−0.150	0.326	0.117	0.909
**Tree cover (%)**	0.955	0.078	−0.108	2.018	1.029	0.996	1.076
**Ground cover (%)**	−1.200	0.232	−3.165	0.766	0.938	0.846	1.041

To explore fourth-order habitat selection, we developed RSFs using 42 dens and 42 paired sites (Table 3). Variables retained after assessment of variation and univariate modeling included soil worm diameter (mm); percent cover by trees, shrubs, herbs, and bare ground; slope (%); hiding cover (%); canopy cover (%); hiding cover variance; and both hiding and canopy cover difference (Supplementary Data SD3). Of these predictors, percent cover by shrubs, herbs, and bare ground; and hiding cover performed best as quadratic terms (Supplementary Data SD4). Correlated terms (*r *> 0.6) included percent shrub and herb cover, and hiding cover variance and difference.

The all-subsets modeling approach resulted in 4 models within 2 AIC_c_ (Table 3; Fig. 3). Model-averaged results suggested that slope had the biggest effect on den likelihood (*β* = 3.373, *P *< 0.001), with each percent increase in slope increasing the odds of a den by 21% (Fig. 3B). Stable soils, as indicated by small soil worm diameters, also predicted dens (*β* = −1.765, *P *= 0.032). For every 1 mm increase in soil worm diameter, the odds of a den decreased by 67% (Fig. 3C). Additionally, for every 10% increase in shrub cover (*β* = −1.342, *P *= 0.025), odds of a den decreased by 35%, Finally, greater percent cover by both trees (*β*  =  0.955, *P *= 0.078) and herbs (*β*  =  1.254, *P *= 0.027) increased odds of a den. For a 10% increase in tree cover and herb cover, odds of a den increased by 29% and 40%, respectively. The models had low multicollinearity (maximum variance inflation factor of 1.62). Models distinguished effectively between used and available sites as indicated by high average AUC ROC (0.951), and high mean Nagelkerke (0.762) and McFadden (0.611) pseudo *R^2^* values suggested a good fit.

**Fig. 3:**
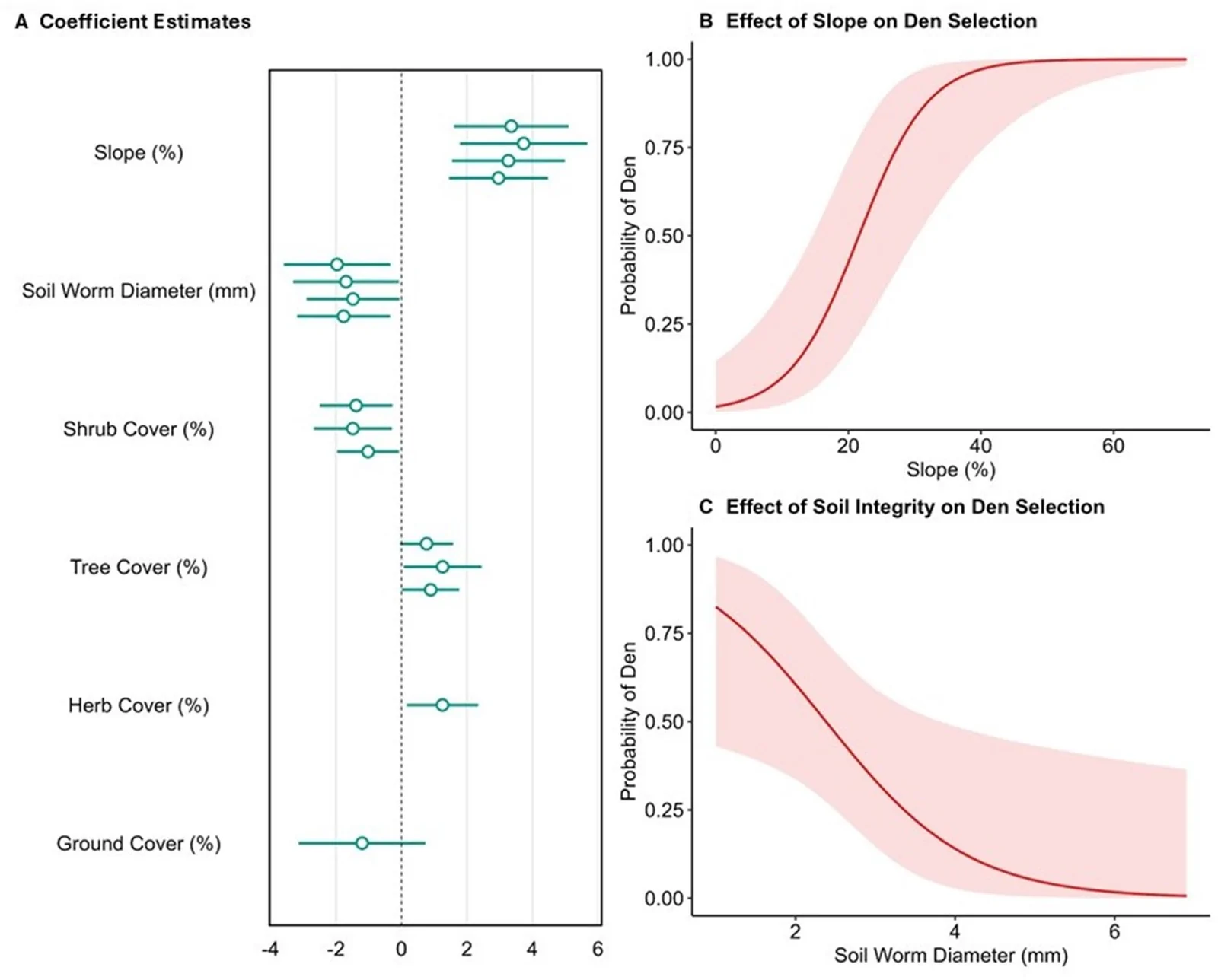
Standardized fourth-order parameter estimates and 95% confidence variables for all models within 2 Akaike’s Information Criterion including key variables selected by coyotes (*Canis latrans*) for den sites in Elk Island National Park, Alberta, Canada, 2024. The parameters are ordered by frequency of occurrence in all models from top to bottom. (A) Coefficient estimates including 95% confidence intervals ordered by frequency of occurrence in all models. (B) Effect of slope (%) on den likelihood showing a substantial increase in likelihood after 10% slope. (C) Effect of soil worm diameter (mm) on den likelihood showing a substantial decrease after 2 mm.

## Discussion

In this study, we explored the third and fourth-order characteristics of Coyote den selection in a protected area in central Alberta, Canada. Coyotes demonstrated strong selection for microhabitat features at the fourth-order, and weaker selection at the third-order. Consistent with our first hypothesis, coyotes selected features associated with den suitability and stability, especially at the fourth-order. Specifically, they chose sites with stable, cohesive soils that contribute to structural stability and steep slopes that facilitate drainage. We found some support for our second hypothesis, predicting selection based on security; in addition to providing drainage, steep slopes can provide security, and dens were widely spaced. We did not find support for our third hypothesis, which predicted selection based on access to resources. More than half of the dens that we found incorporated abandoned Beaver excavations and infrastructure, demonstrating the importance of Beaver modifications to the landscape, even after water recedes. Our findings suggest that wild coyotes in natural settings select specific and predictable microhabitat features at den sites, which enhance den suitability and, perhaps, security. This insight could guide evidence-based management for coyotes and other denning species.

Coyotes exhibited stronger selection for fourth-order habitat characteristics than third-order, which was also observed in a nearby population of urban coyotes (Raymond and St. Clair 2023a) and in wolves (Trapp et al. 2008; Person and Russell 2009). In contrast, grizzly bears select more strongly for third-order features related to security and access to resources at dens (Pigeon et al. 2016), although the functions of bear dens for both thermal and physical protection during hibernation differ from Coyote dens. Most features of den suitability, including soil type and structural support, are inherently fine-scale and are therefore more accurately captured at the fourth-order. By contrast, resource availability occurs at a broader scale (Courbin et al. 2013).

Consistent with our first hypothesis, coyotes showed strong selection for features that promote den suitability, especially at the fourth-order. As observed previously (Althoff 1980; Way et al. 2001; Raymond and St. Clair 2023a), structural support was incorporated into most dens, and 2 dens lacking support and located in sandy soils had partially collapsed. Frequent use of abandoned but enduring Beaver infrastructure (e.g., channels, burrows) could further reflect prioritization of structural stability, though selection of such features could also represent reduced energy expenditure associated with excavation. At the fourth-order, strong selection for features promoting den stability and drainage (e.g., steep slopes and stable and inorganic soils) has likewise been documented at Wolf (Trapp et al. 2008), Grizzly Bear (Pigeon et al. 2016), and Coyote dens (Way et al. 2001). Several studies found selection for Coyote dens with specific aspects, presumably to support thermoregulation (Harrison and Gilbert 1985; Raymond and St. Clair 2023a). We did not find evidence of selection for aspect (Althoff 1980; Way et al. 2001), perhaps suggesting that aspect interacts with other microtopography features to promote thermoregulation. At the third-order, avoidance of areas with high water cover likely increases den suitability by limiting the risk of flooding. In the EINP ecosystem, abundant water, flat topography, and frequent organic soils (67% of available sites) may increase the relative importance of selection for criteria supporting den integrity.

We found mixed evidence for our second hypothesis predicting selection for den features that promoted security, though previous studies of coyotes in both rural (Althoff 1979) and urban areas (Way et al. 2001; Raymond and St. Clair 2023a) demonstrate strong selection for such features, as they do for other canids (Trapp et al. 2008; Zaman et al. 2020). At the fourth-order, selection for steep slopes could limit access and detection by people and predators (Raymond and St. Clair 2023a), but steep slopes are also associated with drainage (Trapp et al. 2008). We did not detect selection for hiding cover, which was the most strongly selected fourth-order feature among Coyote dens in a nearby urban environment (Raymond and St. Clair 2023a), though selection for high percent cover by both trees and grass may indicate selection for features that promote security. Among third-order characteristics, if neighboring dens belonged to the same Coyote pair, wide spacing between dens may have promoted security by ensuring that, in the case of a disturbance, alternative dens would be far from a prospective threat (Camenzind 1978). Alternately, if neighboring dens belonged to different Coyote pairs, wide separation would have promoted security from potentially dangerous conspecifics.

Failure to demonstrate strong selection for security features, especially those related to people and their infrastructure, could reflect the local risks that denning coyotes face; EINP provides a relatively safe environment through fenced areas that limit access by people and off-leash dogs, and where no legal persecution of coyotes occurs (Pruss 2002). Wolves prey on Coyote pups, but they are rare in EINP (Foca and Boyce 2022). By contrast, in nearby agricultural areas coyotes are persecuted (Pruss 2002), and in urban environments coyotes have conflicts with humans and dogs (Farr et al. 2023; Raymond and St. Clair 2023b). Flexible denning strategies in response to local environmental pressures have been documented in some species. For example, wolverines (*Gulo gulo*) typically select den features that promote security, but those living in areas with reduced roads and fewer predators selected most strongly for dens that promoted thermoregulation (Jokinen et al. 2019). In other species such as African wild dogs, selection for security persists regardless of predator density (van der Meer et al. 2013), even at the expense of resource access.

We found no evidence that coyotes selected for access to the resources for which we tested (i.e., water or meadow habitat), and coyotes avoided both proximity to water and water cover at the third-order. Water avoidance contradicts previous studies in which coyotes (Way et al. 2001; Raymond and St. Clair 2023a) and wolves (Trapp et al. 2008; Person and Russell 2009) selected for water proximity, presumably to support hydration in lactating females. In EINP, where water is abundant, immediate water proximity may be unnecessary, especially in spring when pups are born and snowmelt results in extensive ephemeral flooding (Hood 2007). We also found no selection for vegetation associated with small mammal prey, contrasting other studies (Althoff 1980; Ward et al. 2018). Although females rarely leave the den in the days following birth (Ryon 1986), males are thought to supply food for the mother, limiting the need for immediate prey access (Harrison and Gilbert 1985; Way et al. 2001). Because coyotes are highly mobile predators that have variable and diverse diets influenced by season and habitat (Sugden et al. 2021; Raymond et al. 2024), immediate access to small mammal hunting opportunities may be trivial.

This study provides insight into the habitat characteristics selected by wild coyotes in natural settings at dens, but some limitations may affect the generalizability of our results. First, we could not quantify predator abundance, which may be an important predictor in resource selection (DeCesare et al. 2014), nor could we assess recency of use for pup-rearing or den success. Second, campsites, buildings, and trails were sufficiently infrequent in EINP that we did not include these metrics as predictors, but they may have, nonetheless, influenced selection (Malcolm et al. 2020). Third, for our fourth-order analysis, we used a single available site per use site, which could have limited our assessment of microsite variability. This ratio has been used in several studies of fine-scale habitat selection (Klaassen and Broekhuis 2018; Schweiger et al. 2021), and our multiple metrics of model performance all suggested that our findings were robust. Additionally, we could not be sure that the dens we detected had been used as natal dens. Finally, to avoid disturbing pups, we measured den characteristics outside of the obligate denning period and 1 yr after first finding dens. Because Coyote dens are often used year after year (authors’ personal observations), sometimes for decades (Crabtree and Sheldon 1999), we suspect that this did not negatively influence our findings.

Our results demonstrate that coyotes select strongly for fine-scale habitat characteristics, especially those that promote den suitability and stability (Table 1). This finding contrasts other studies which have documented prioritization of security features above others (Althoff 1979:197; Way et al. 2001; Raymond and St. Clair 2023a), perhaps owing to relative safety from people, predators, and dogs. Flexible selection in response to different local environmental pressures reflects the adaptability and behavioral plasticity that have enabled coyotes to expand their range and occupy a diversity of landscapes (Breck et al. 2019; Sugden et al. 2021). To better inform adaptive management strategies, future studies could explore how den selection by coyotes and other species changes under the influence of varying ecological pressures in diverse landscapes.

## Supplementary Material

gyag072_Supplementary_Data

## Data Availability

The code used to perform the analysis and develop the figures in this manuscript is available at: https://github.com/sageraymond/WildDenSelection

## Supplementary data

**Supplementary Data SD1**. Summary information for univariate models explaining third-order characteristics selected by coyotes (*Canis latrans*) at den sites and available sites in Elk Island National Park, Alberta, Canada, 2024. All predictors were included as linear terms. Metrics reported include sample size (*n*), Akaike Information Criterion adjusted for small sample sizes (AICc), standardized beta coefficients (*β*), *P* values (*P*), upper and lower confidence intervals (CI), and McFadden (McF.) and Nagelkerke (Nag.) pseudo *R2*.

**Supplementary Data SD2**. Summary information for univariate models explaining third-order characteristics selected by coyotes (*Canis latrans*) at den sites and available sites in Elk Island National Park, Alberta, Canada, 2024. All predictors were included as quadratic terms. Metrics reported include sample size (*n*), Akaike Information Criterion adjusted for small sample sizes (AICc), standardized beta coefficients (*β*), *P* values (*P*), upper and lower confidence intervals (CI), and McFadden (McF.) and Nagelkerke (Nag.) pseudo *R2*.

**Supplementary Data SD3**. Summary information for univariate ­models explaining fourth-order characteristics selected by coyotes (*Canis latrans*) at den sites and available sites in Elk Island National Park, Alberta, Canada, 2024. All predictors were included as linear terms. Metrics reported include sample size (*n*), Akaike Information Criterion adjusted for small sample sizes (AICc), standardized beta coefficients (*β*), *P* values (*P*), upper and lower confidence intervals (CI), and McFadden (McF.) and Nagelkerke (Nag.) pseudo *R2*.

**Supplementary Data SD4**. Summary information for univariate models explaining fourth-order characteristics selected by coyotes (*Canis latrans*) at den sites and available sites in Elk Island National Park, Alberta, Canada, 2024. All predictors were included as quadratic terms. Metrics reported include sample size (*n*), Akaike Information Criterion adjusted for small sample sizes (AICc), standardized beta coefficients (*β*), *P* values (*P*), upper and lower confidence intervals (CI), and McFadden (McF.) and Nagelkerke (Nag.) pseudo *R2*.
