# Supplementary material for: Denning coyotes (Canis latrans) select fine-scale habitat characteristics that promote den stability in a protected area
Source: J Mammal. 2026 Aug 19;107(5):1124–34. doi: 10.1093/jmammal/gyag072 (PMC13722707; doi:10.1093/jmammal/gyag072)
Supplement: Supplementary file 1 [file gyag072_supplementary_data.zip › Supplementary Data SD1.docx]

**Supplementary Data**

**Supplementary Data SD1**. Summary information for univariate models explaining third-order characteristics selected by coyotes (*Canis latrans*) at den sites and available sites in Elk Island National Park, Alberta, Canada, 2024. All predictors were included as linear terms. Metrics reported include sample size (*n*), Akaike Information Criterion adjusted for small sample sizes (AIC_c_), standardized beta coefficients (*β*), *P* values (*P*), upper and lower confidence intervals (CI), and McFadden (McF.) and Nagelkerke (Nag.) pseudo *R^2^*.

| Variable | *n* | AIC_c_ | *β* | *P* | Low CI | Upp CI | McF. | Nag. |
| --- | --- | --- | --- | --- | --- | --- | --- | --- |
| Dist. to Trees (m) | 252 | 213.7 | 2.764 | 0.004 | 1.132 | 4.881 | 0.077 | 0.112 |
| Dist. to Open (m) | 252 | 212.3 | 2.433 | 0.005 | 1.007 | 4.360 | 0.083 | 0.121 |
| Slope (%) | 252 | 229.3 | 0.239 | 0.199 | -0.109 | 0.625 | 0.008 | 0.012 |
| Elevation (m) | 252 | 230.9 | 0.082 | 0.638 | -0.246 | 0.444 | 0.001 | 0.002 |
| Open Edge Density (m/km^2^) | 252 | 226.5 | -0.374 | 0.035 | -0.732 | -0.033 | 0.020 | 0.031 |
| Tree Edge Density (m/km^2^) | 252 | 230.7 | -0.106 | 0.533 | -0.441 | 0.227 | 0.002 | 0.003 |
| Tree Cover 12.5 (%) | 252 | 230.9 | -0.064 | 0.709 | -0.404 | 0.268 | 0.001 | 0.001 |
| Tree Cover 25 (%) | 252 | 230.5 | 0.128 | 0.446 | -0.204 | 0.457 | 0.003 | 0.004 |
| Tree Cover 50 (%) | 252 | 226.7 | 0.354 | 0.038 | 0.023 | 0.693 | 0.019 | 0.029 |
| Tree Cover 100 (%) | 252 | 225.5 | 0.405 | 0.019 | 0.070 | 0.751 | 0.025 | 0.037 |
| Tree Cover 250 (%) | 252 | 229.7 | 0.194 | 0.246 | -0.136 | 0.523 | 0.006 | 0.009 |
| Tree Cover 500 (%) | 252 | 230.7 | -0.111 | 0.520 | -0.461 | 0.221 | 0.002 | 0.003 |
| Water Cover 12.5 (%) | 252 | 207.8 | -0.674 | <0.001 | -1.006 | -0.392 | 0.103 | 0.149 |
| Water Cover 25 (%) | 252 | 203.1 | -0.751 | <0.001 | -1.073 | -0.465 | 0.123 | 0.177 |
| Water Cover 50 (%) | 252 | 199.0 | -0.820 | <0.001 | -1.132 | -0.532 | 0.141 | 0.202 |
| Water Cover 100 (%) | 252 | 202.9 | -0.800 | <0.001 | -1.116 | -0.502 | 0.124 | 0.178 |
| Water Cover 250 (%) | 252 | 217.2 | -0.579 | 0.000 | -0.888 | -0.277 | 0.061 | 0.090 |
| Water Cover 500 (%) | 252 | 230.9 | -0.072 | 0.664 | -0.388 | 0.265 | 0.001 | 0.001 |
| Open Cover 12.5 (%) | 252 | 230.1 | 0.171 | 0.342 | -0.168 | 0.544 | 0.004 | 0.006 |
| Open Cover 25 (%) | 252 | 230.0 | 0.186 | 0.306 | -0.155 | 0.566 | 0.005 | 0.007 |
| Open Cover 50 (%) | 252 | 229.5 | 0.227 | 0.223 | -0.120 | 0.618 | 0.007 | 0.011 |
| Open Cover 100 (%) | 252 | 228.8 | 0.281 | 0.148 | -0.076 | 0.691 | 0.010 | 0.015 |
| Open Cover 250 (%) | 252 | 225.0 | 0.473 | 0.022 | 0.091 | 0.909 | 0.027 | 0.040 |
| Open Cover 500 (%) | 252 | 227.6 | 0.345 | 0.075 | -0.015 | 0.750 | 0.015 | 0.023 |
| Road Density (m/km^2^) | 252 | 223.9 | 0.598 | 0.023 | 0.144 | 1.192 | 0.032 | 0.048 |
| East Index | 252 | 226.1 | 0.372 | 0.026 | 0.046 | 0.702 | 0.022 | 0.033 |
| South Index | 252 | 229.8 | 0.193 | 0.246 | -0.136 | 0.519 | 0.006 | 0.009 |
| Dec. Dist. to Den | 252 | 185.5 | 1.043 | <0.001 | 0.729 | 1.384 | 0.201 | 0.278 |
| Dec. Dist. to Water | 252 | 199.2 | 0.799 | <0.001 | 0.520 | 1.100 | 0.140 | 0.200 |
| Dec. Dist. to Park Boundary | 252 | 226.4 | -0.524 | 0.081 | -1.261 | -0.042 | 0.021 | 0.031 |
