# Supplementary material for: Denning coyotes (Canis latrans) select fine-scale habitat characteristics that promote den stability in a protected area
Source: J Mammal. 2026 Aug 19;107(5):1124–34. doi: 10.1093/jmammal/gyag072 (PMC13722707; doi:10.1093/jmammal/gyag072)
Supplement: Supplementary file 1 [file gyag072_supplementary_data.zip › Supplementary Data SD2.docx]

**Supplementary Data**

**Supplementary Data SD2**. Summary information for univariate models explaining third-order characteristics selected by coyotes (*Canis latrans*) at den sites and available sites in Elk Island National Park, Alberta, Canada, 2024. All predictors were included as quadratic terms. Metrics reported include sample size (*n*), Akaike Information Criterion adjusted for small sample sizes (AIC_c_), standardized beta coefficients (*β*), *P* values (*P*), upper and lower confidence intervals (CI), and McFadden (McF.) and Nagelkerke (Nag.) pseudo *R^2^*.

|  |  |  | Quadratic | | | |  | Linear | | | |  |  |
| --- | --- | --- | --- | --- | --- | --- | --- | --- | --- | --- | --- | --- | --- |
| Variable | *n* | AIC_c_ | *β* | *P* | Low CI | Upp CI |  | *β* | *P* | Low CI | Upp CI | McF. | Nag. |
| Dist. to Trees (m) | 252 | 213.2 | 7.833 | 0.199 | 0.815 | 22.187 |  | 5.771 | 0.057 | 1.672 | 13.483 | 0.087 | 0.127 |
| Dist. to Open (m) | 252 | 211.9 | 5.885 | 0.214 | 0.433 | 17.355 |  | 5.021 | 0.070 | 1.418 | 12.322 | 0.093 | 0.136 |
| Slope (%) | 252 | 231.3 | -0.009 | 0.941 | -0.228 | 0.285 |  | 0.246 | 0.233 | -0.162 | 0.651 | 0.008 | 0.012 |
| Elevation (m) | 252 | 222.3 | -0.253 | 0.001 | -0.404 | -0.104 |  | 0.393 | 0.027 | 0.043 | 0.747 | 0.048 | 0.071 |
| Open Edge Density (m/km^2^) | 252 | 224.7 | 0.341 | 0.087 | -0.002 | 0.786 |  | -0.512 | 0.028 | -1.018 | -0.087 | 0.037 | 0.055 |
| Tree Edge Density (m/km^2^) | 252 | 230.3 | 0.210 | 0.169 | -0.046 | 0.563 |  | -0.152 | 0.457 | -0.572 | 0.243 | 0.012 | 0.019 |
| Tree Cover 12.5 (%) | 252 | 228.8 | 0.516 | 0.042 | 0.020 | 1.019 |  | 0.058 | 0.765 | -0.323 | 0.448 | 0.019 | 0.029 |
| Tree Cover 25 (%) | 252 | 213.2 | 0.976 | <0.001 | 0.515 | 1.508 |  | 0.546 | 0.034 | 0.066 | 1.091 | 0.088 | 0.128 |
| Tree Cover 50 (%) | 252 | 199.0 | 1.349 | <0.001 | 0.781 | 2.068 |  | 1.100 | 0.002 | 0.483 | 1.879 | 0.150 | 0.213 |
| Tree Cover 100 (%) | 252 | 199.3 | 1.378 | 0.000 | 0.764 | 2.184 |  | 1.105 | 0.002 | 0.483 | 1.888 | 0.149 | 0.211 |
| Tree Cover 250 (%) | 252 | 215.9 | 0.780 | 0.002 | 0.348 | 1.344 |  | 0.492 | 0.057 | 0.002 | 1.035 | 0.076 | 0.111 |
| Tree Cover 500 (%) | 252 | 218.4 | 0.853 | 0.003 | 0.353 | 1.492 |  | 0.004 | 0.988 | -0.516 | 0.514 | 0.065 | 0.096 |
| Water Cover 12.5 (%) | 252 | 206.7 | 0.201 | 0.068 | -0.023 | 0.423 |  | -1.436 | 0.002 | -2.367 | -0.543 | 0.116 | 0.168 |
| Water Cover 25 (%) | 252 | 200.0 | 0.234 | 0.023 | 0.032 | 0.455 |  | -1.507 | <0.001 | -2.276 | -0.789 | 0.146 | 0.207 |
| Water Cover 50 (%) | 252 | 200.6 | 0.075 | 0.560 | -0.201 | 0.314 |  | -0.996 | 0.004 | -1.659 | -0.307 | 0.143 | 0.203 |
| Water Cover 100 (%) | 252 | 202.5 | -0.240 | 0.152 | -0.612 | 0.056 |  | -0.394 | 0.204 | -0.990 | 0.239 | 0.135 | 0.193 |
| Water Cover 250 (%) | 252 | 213.2 | -0.401 | 0.031 | -0.806 | -0.072 |  | -0.038 | 0.891 | -0.574 | 0.528 | 0.087 | 0.128 |
| Water Cover 500 (%) | 252 | 228.8 | 0.377 | 0.061 | 0.012 | 0.813 |  | -0.428 | 0.084 | -0.920 | 0.054 | 0.019 | 0.029 |
| Open Cover 12.5 (%) | 252 | 230.9 | -0.301 | 0.268 | -0.859 | 0.217 |  | 0.441 | 0.151 | -0.135 | 1.082 | 0.010 | 0.015 |
| Open Cover 25 (%) | 252 | 231.0 | -0.206 | 0.316 | -0.614 | 0.200 |  | 0.385 | 0.156 | -0.134 | 0.934 | 0.009 | 0.014 |
| Open Cover 50 (%) | 252 | 229.7 | -0.242 | 0.172 | -0.588 | 0.119 |  | 0.478 | 0.072 | -0.031 | 1.017 | 0.015 | 0.022 |
| Open Cover 100 (%) | 252 | 226.7 | -0.319 | 0.038 | -0.624 | -0.010 |  | 0.656 | 0.015 | 0.142 | 1.202 | 0.028 | 0.042 |
| Open Cover 250 (%) | 252 | 221.2 | -0.383 | 0.010 | -0.676 | -0.078 |  | 0.790 | 0.001 | 0.333 | 1.277 | 0.052 | 0.078 |
| Open Cover 500 (%) | 252 | 229.6 | -0.008 | 0.963 | -0.337 | 0.386 |  | 0.351 | 0.118 | -0.086 | 0.797 | 0.015 | 0.023 |
| Road Density (m/km^2^) | 252 | 215.0 | 0.915 | 0.002 | 0.415 | 1.574 |  | -0.511 | 0.065 | -1.081 | 0.022 | 0.080 | 0.116 |
| East Index | 252 | 225.1 | -0.184 | 0.334 | -0.533 | 0.309 |  | 0.860 | 0.035 | 0.111 | 1.752 | 0.035 | 0.053 |
| South Index | 252 | 227.4 | -0.196 | 0.402 | -0.663 | 0.261 |  | 0.261 | 0.210 | -0.137 | 0.684 | 0.025 | 0.038 |
| Dec. Dist. to Den | 252 | 231.5 | 0.109 | 0.607 | -0.304 | 0.530 |  | 0.251 | 0.218 | -0.138 | 0.664 | 0.007 | 0.011 |
| Dec. Dist. to Water | 252 | 187.5 | 0.030 | 0.857 | -0.305 | 0.350 |  | 1.094 | 0.001 | 0.455 | 1.756 | 0.201 | 0.279 |
| Dec. Dist. to Park Boundary | 252 | 201.2 | 0.040 | 0.804 | -0.291 | 0.352 |  | 0.900 | 0.039 | 0.023 | 1.745 | 0.141 | 0.200 |
