# Supplementary material for: Denning coyotes (Canis latrans) select fine-scale habitat characteristics that promote den stability in a protected area
Source: J Mammal. 2026 Aug 19;107(5):1124–34. doi: 10.1093/jmammal/gyag072 (PMC13722707; doi:10.1093/jmammal/gyag072)
Supplement: Supplementary file 1 [file gyag072_supplementary_data.zip › Supplementary Data SD3.docx]

**Supplementary Data**

**Supplementary Data SD3**. Summary information for univariate models explaining fourth-order characteristics selected by coyotes (*Canis latrans*) at den sites and available sites in Elk Island National Park, Alberta, Canada, 2024. All predictors were included as linear terms. Metrics reported include sample size (*n*), Akaike Information Criterion adjusted for small sample sizes (AIC_c_), standardized beta coefficients (*β*), *P* values (*P*), upper and lower confidence intervals (CI), and McFadden (McF.) and Nagelkerke (Nag.) pseudo *R^2^*.

| Variable | *n* | AIC_c_ | *β* | *P* | Low CI | Upp CI | McF. | Nag. |
| --- | --- | --- | --- | --- | --- | --- | --- | --- |
| Min. Soil Diameter (mm) | 84 | 83.9 | -2.110 | <0.001 | -3.248 | -1.236 | 0.314 | 0.470 |
| Perc. Tree | 84 | 118.9 | 0.276 | 0.214 | -0.156 | 0.722 | 0.013 | 0.025 |
| Perc. Shrub | 84 | 118.7 | 0.291 | 0.194 | -0.143 | 0.741 | 0.015 | 0.027 |
| Perc. Herb | 84 | 119.5 | -0.214 | 0.333 | -0.656 | 0.217 | 0.008 | 0.015 |
| Perc. Ground | 84 | 119.2 | 0.250 | 0.277 | -0.187 | 0.736 | 0.011 | 0.020 |
| East Index | 79 | 112.5 | 0.193 | 0.398 | -0.253 | 0.650 | 0.007 | 0.012 |
| South Index | 79 | 112.4 | -0.209 | 0.362 | -0.665 | 0.237 | 0.008 | 0.014 |
| Avg. Slope (%) | 84 | 66.2 | 2.655 | <0.001 | 1.711 | 3.901 | 0.466 | 0.635 |
| Avg. Canopy Cover (%) | 84 | 120.0 | -0.152 | 0.491 | -0.595 | 0.279 | 0.004 | 0.008 |
| Canopy Cover Variance | 83 | 118.7 | 0.130 | 0.559 | -0.305 | 0.580 | 0.003 | 0.006 |
| Canopy Cover Difference | 83 | 119.0 | 0.044 | 0.844 | -0.392 | 0.482 | 0.000 | 0.001 |
| Avg. Hiding Cover | 84 | 109.7 | -0.776 | 0.002 | -1.306 | -0.302 | 0.093 | 0.161 |
| Hiding Cover Variance | 83 | 101.6 | 1.089 | 0.000 | 0.542 | 1.736 | 0.152 | 0.253 |
| Hiding Cover Difference | 83 | 101.7 | 1.038 | 0.000 | 0.523 | 1.630 | 0.151 | 0.252 |
