# Supplementary material for: Denning coyotes (Canis latrans) select fine-scale habitat characteristics that promote den stability in a protected area
Source: J Mammal. 2026 Aug 19;107(5):1124–34. doi: 10.1093/jmammal/gyag072 (PMC13722707; doi:10.1093/jmammal/gyag072)
Supplement: Supplementary file 1 [file gyag072_supplementary_data.zip › Supplementary Data SD4.docx]

**Supplementary Data**

**Supplementary Data SD4**. Summary information for univariate models explaining fourth-order characteristics selected by coyotes (*Canis latrans*) at den sites and available sites in Elk Island National Park, Alberta, Canada, 2024. All predictors were included as quadratic terms. Metrics reported include sample size (*n*), Akaike Information Criterion adjusted for small sample sizes (AIC_c_), standardized beta coefficients (*β*), *P* values (*P*), upper and lower confidence intervals (CI), and McFadden (McF.) and Nagelkerke (Nag.) pseudo *R^2^*.

|  |  |  | Quadratic | | | |  | Linear | | | |  |  |
| --- | --- | --- | --- | --- | --- | --- | --- | --- | --- | --- | --- | --- | --- |
| Variable | *n* | AIC_c_ | *β* | *P* | Low CI | Upp CI |  | *β* | *P* | Low CI | Upp CI | McF. | Nag. |
| Min. Soil Diameter (mm) | 84 | 85.6 | -0.389 | 0.610 | -2.204 | 0.684 |  | -2.207 | <0.001 | -3.791 | -1.222 | 0.316 | 0.473 |
| Perc. Tree | 84 | 120.8 | 0.074 | 0.821 | -0.560 | 0.734 |  | 0.258 | 0.278 | -0.205 | 0.731 | 0.014 | 0.025 |
| Perc. Shrub | 84 | 96.7 | -1.359 | <0.001 | -2.054 | -0.769 |  | 0.450 | 0.118 | -0.095 | 1.053 | 0.221 | 0.351 |
| Perc. Herb | 84 | 97.0 | -1.638 | <0.001 | -2.440 | -0.942 |  | -0.255 | 0.337 | -0.793 | 0.262 | 0.218 | 0.348 |
| Perc. Ground | 84 | 116.4 | -0.351 | 0.036 | -0.713 | -0.037 |  | 0.900 | 0.019 | 0.168 | 1.685 | 0.052 | 0.093 |
| East Index | 84 | 114.2 | -0.175 | 0.596 | -0.830 | 0.474 |  | 0.221 | 0.346 | -0.236 | 0.688 | 0.009 | 0.017 |
| South Index | 79 | 114.1 | 0.155 | 0.635 | -0.489 | 0.805 |  | -0.208 | 0.364 | -0.666 | 0.239 | 0.010 | 0.018 |
| Avg. Slope (%) | 79 | 67.2 | -0.547 | 0.258 | -1.336 | 0.656 |  | 2.670 | <0.001 | 1.754 | 3.873 | 0.475 | 0.643 |
| Avg. Canopy Cover (%) | 84 | 122.0 | -0.004 | 0.986 | -0.490 | 0.483 |  | -0.155 | 0.596 | -0.740 | 0.420 | 0.004 | 0.008 |
| Canopy Cover Variance | 84 | 120.0 | 0.199 | 0.411 | -0.260 | 0.713 |  | -0.148 | 0.712 | -0.945 | 0.636 | 0.009 | 0.017 |
| Canopy Cover Difference | 83 | 120.6 | 0.150 | 0.537 | -0.323 | 0.641 |  | -0.060 | 0.829 | -0.609 | 0.485 | 0.004 | 0.007 |
| Avg. Hiding Cover | 83 | 110.6 | -0.242 | 0.309 | -0.720 | 0.228 |  | -0.818 | 0.002 | -1.368 | -0.331 | 0.101 | 0.175 |
| Hiding Cover Variance | 84 | 102.2 | -0.291 | 0.192 | -0.708 | 0.238 |  | 1.290 | <0.001 | 0.642 | 2.017 | 0.164 | 0.270 |
| Hiding Cover Difference | 83 | 103.3 | -0.163 | 0.555 | -0.721 | 0.378 |  | 1.060 | <0.001 | 0.537 | 1.672 | 0.154 | 0.256 |
